# Adjustable breathing resistance for laryngectomized patients: Proof of principle in a novel heat and moisture exchanger cassette

**DOI:** 10.1002/hed.26571

**Published:** 2020-12-08

**Authors:** Maartje Leemans, Sara H. Muller, Maarten J. A. van Alphen, Wim Vallenduuk, Richard Dirven, Michiel W. M. van den Brekel

**Affiliations:** ^1^ Department of Head and Neck Oncology and Surgery The Netherlands Cancer Institute – Antoni van Leeuwenhoek Amsterdam The Netherlands; ^2^ Department of Clinical Physics and Instrumentation The Netherlands Cancer Institute – Antoni van Leeuwenhoek Amsterdam The Netherlands; ^3^ Institute of Phonetic Sciences University of Amsterdam Amsterdam The Netherlands; ^4^ Department of Oral and Maxillofacial Surgery Amsterdam University Medical Center (AUMC) Amsterdam The Netherlands

**Keywords:** breathing resistance, heat and moisture exchanger, HME cassette, pulmonary rehabilitation, total laryngectomy

## Abstract

**Background:**

Due to the heat and moisture exchanger's (HME) breathing resistance, laryngectomized patients cannot always use an (optimal) HME during physical exercise. We propose a novel HME cassette concept with adjustable “bypass,” to provide adjustment between different breathing resistances within one device.

**Methods:**

Under standardized conditions, the resistance and humidification performance of a high resistance/high humidification HME (XM) foam in a cassette with and without bypass were compared to a lower resistance/lesser humidification HME (XF) foam in a closed cassette.

**Results:**

With a bypass in the cassette, the resistance and humidification performance of XM foam were similar to those of XF foam in the closed cassette. Compared to XM foam in the closed cassette, introducing the bypass resulted in a 40% resistance decrease, whereas humidification performance was maintained at 80% of the original value.

**Conclusions:**

This HME cassette prototype allows adjustment between substantially different resistances while maintaining appropriate humidification performances.

## 1 INTRODUCTION

Heat and moisture exchangers (HMEs) are used as a standard treatment for pulmonary rehabilitation after a total laryngectomy.^1^, ^2^, ^3^, ^4^, ^5^ Normally, the upper airways condition (heat and humify) the inhaled air, but in laryngectomized patients the lungs are exposed to the dry and cold air during open stoma breathing. An HME covering the stoma can to some extent improve the pulmonary condition. The benefits of HME use have been underlined in many studies; it does not only improve the pulmonary functioning, such as a decrease in mucus production, coughing, and forced expectorations, but also the psychosocial functioning of laryngectomized patients.^1^, ^2^, ^3^, ^4^, ^6^, ^7^, ^8^ Laryngectomized patients are recommended to continuously use an HME with the highest possible humidification performance (the highest water exchange).^9^, ^10^

The humidification performance of the HME, and thus its benefits, rely mainly on the HME core material and cassette design. The HME core material often consists of a porous polymer foam impregnated with hygroscopic salt, which acts as a condensation and evaporation surface.^11^, ^12^, ^13^ Since the HME is a passive humidifier, its humidification performance can primarily be improved by increasing the width and height of the core material or decreasing the foam's pore size. Increase of width and height are limited by aesthetic considerations. Additionally, these performance improvements have a trade off with the HME's breathing resistance and consequently patient acceptance. To cater to the different patient needs and activity levels, multiple types of HMEs have been developed, which vary in resistance and performance.^9^, ^14^

Nevertheless, complete HME compliance has not yet been achieved in all laryngectomized patients. Laryngectomized patients discontinue their (high humidification performance) HME use due to the higher breathing resistance of the HME compared to open stoma breathing, especially periodically during physical activities.^1^, ^10^, ^15^, ^16^, ^17^, ^18^, ^19^ Other reasons for laryngectomized patients to discontinue their HME use, outside the scope of this study, include: adhesive related skin irritation, mucus problems or the HME's aesthetics.^1^, ^9^, ^14^, ^15^, ^17^, ^19^ Although physical exercise can sometimes be anticipated, changing between different HME types with varying breathing resistance is not always an option or requires additional effort and preparation.^1^, ^2^ As a result, some patients do not use any HME at all.

Patient compliance and comfort during different levels of physical activities could potentially be improved by providing one HME device that enables a quick and simple adjustment of the breathing resistance based on the patient's activity level. During rest, a laryngectomized patient can use the HME device with a higher resistance and humidification performance setting. Alternatively, during physical activities the HME device can be adjusted to decrease its resistance, while maintaining an appropriate humidification performance.

We propose a novel HME cassette concept with an adjustable “bypass” at its base. In this study, we designed and tested this adjustable HME cassette prototype to validate that it will result in substantially different breathing resistances with appropriate humidification performances for each level of activity.

## 2 MATERIALS AND METHODS

### 2.1 HME devices and prototype

**FIGURE 1:**
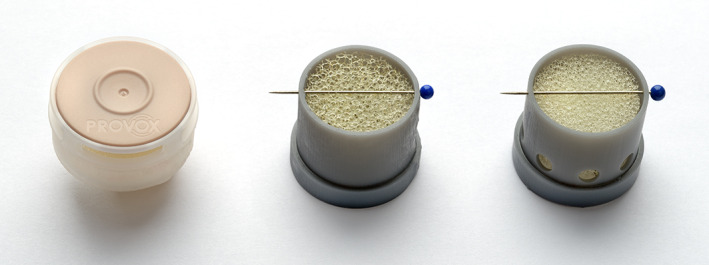
The photo shows, from left to right, the original HME cassette of both the XF and XM with speaking valve (pink lid), the 3D‐printed (FormLabs, Form2) closed cassette with inserted XF foam and the 3D‐printed (FormLabs, Form2) cassette with bypass on the tracheal side, with inserted XM foam (note the difference in pore size between the two different foams). A speaking valve was not included in the 3D printed cassette designs to simplify the prototyping and to limit the scope of this proof of principle study to only the effect of the bypass. The thicker cylinder at the base of the 3D‐printed cassettes is used to connect them to the measurement set‐up (spirometer). HME, heat and moisture exchanger; XF, lower resistance/lesser humidification HME; XM, high resistance/high humidification HME [Color figure can be viewed at wileyonlinelibrary.com]

In this study, we used two types of HME foams taken from the two most commonly used HMEs at the Netherlands Cancer Institute – Antoni van Leeuwenhoek: the Provox^®^ XtraMoist^TM^ HME (XM) and the Provox^®^ XtraFlow^TM^ HME (XF, both Atos Medical AB, Malmö, Sweden). An overview of the specifications of the Pressure Drop and Moisture Loss, and of the measurements of the Water Exchange of the XM and XF are given in Table 1. Water Exchange is a direct measure of the humidification performance.^22^ The XM is one of the highest performing commonly used HMEs.^14^ The XF is considered to be an HME with an “acceptable” breathing resistance by the majority of the laryngectomized patients, unable to (continuously) tolerate the higher breathing resistance of the XM.^1^, ^10^ However, the XF has a lesser humidification performance compared to the XM. The HME cassettes of the XM and XF are identical: the differences in breathing resistance and performance are due to the difference in core material (Figure 1).

**TABLE 1:** Specifications of the Moisture Loss and Pressure Drop values of the Provox XtraMoist (XM) and Provox XtraFlow (XF), as provided by the manufacturer (Atos Medical AB, Malmö, Sweden) in accordance with ISO 9360‐2:2001,^21^ and the humidification performance (Water Exchange) as reported by previous studies.

	Pressure Drop (Pa)			Moisture Loss^a^ (mg/L)	Water Exchange (mg) Van den Boer et al. (2014a)^14^	Water Exchange (mg) Van den Boer et al. (2014b)^21^
HME	At 30 L/min	At 60 L/min	At 90 L/min	At *V*_*T*_ = 1 L (*AH_amb‐ref_* = 0 mg/L)	At *V*_*T*_ = 0.5 L (*AH_amb‐ref_* = 5 mg/L)	At *V*_*T*_ = 0.5 L (*AH_amb‐ref_* = 5 mg/L)
Provox XtraMoist	70	240	480	21.5	3.61	3.63
Provox XtraFlow	40	130	290	24.0	2.89	1.95

*Note*: The pressure drop of the XF at a flow of 60 L/min is approximately 60% of that of the XM. The humidification performance (Water Exchange) of the XF shows relatively less decline: approximately 80% of that of the XM.

Abbreviations: *AH_amb‐ref_*, chosen reference value for ambient humidity; HME, heat and moisture exchanger; ISO, International Organization for Standardization; *V_T_*, tidal volume.

^a^ The lower the moisture loss value, the better the HME's humidification performance.

In this study, we use the pressure drop as a measure for resistance (in Appendix A, the mathematical relationship between pressure drop, flow and resistance can be found). Water Exchange, the amount of water an HME evaporates during inhalation and condensates during exhalation, is used as a measure of humidification performance.^14^

The high breathing resistance of an HME can be reduced by introducing a relatively simple “bypass” in the HME cassette, or a simple hole in the HME foam (see Appendix A). A bypass functions as a “shortcut” for the airflow and will therefore decrease both resistance and humidification performance. Due to the almost quadratic relationship between flow and resistance (Appendix A), a bypass reduces the HME's breathing resistance considerably more than its humidification performance.

**FIGURE 2:**
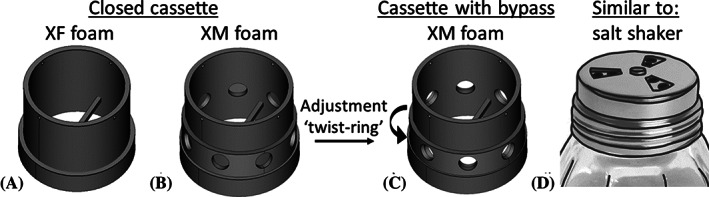
The two HME cassette types. A, Design of the closed cassette for the XF foam measurements. B, Design of the closed cassette for the XM foam measurements. The bypass on the tracheal side of the cassette is closed off with a “twist‐ring.” C, 3D‐design of the cassette with opened bypass for the XM foam measurements. The specific bypass consists of eight *d* = 4 mm holes at the base of the cassette and can be opened or closed by adjusting the “twist‐ring.” D, “Twist‐ring” concept as seen on salt shakers. The bar at the base and the two small holes at the top of the cassettes, intended for inserting a pin, keep the HME foam in place during the measurements. The thicker cylinder at the base of the 3D‐printed cassettes is used to connect them to the measurement set‐up (spirometer). HME, heat and moisture exchanger; XF, lower resistance/lesser humidification HME; XM, high resistance/high humidification HME

A bypass should be designed which can easily be opened or closed and does not interfere with the HME's speaking valve. Additionally, it is desirable that this specific bypass can modify an XM‐like HME into an HME with the properties comparable to an XF. Therefore, the following 3D‐printed (FormLabs, Form2) HME cassette designs were used as a prototype in this study: two simplified closed straight cylindrical cassettes without a speaking valve, Figure 2a,b (further on called the “closed cassette”‐type), and a similar cassette with an opened bypass at its tracheal side, Figure 2c (further on called the “cassette with bypass”‐type). The bypass consists of eight holes with a diameter of 4 mm, distributed evenly around the cassette's base, which can quickly and easily be opened or closed by adjusting a “twist‐ring” (compare Figure 2b and 2c, similar to the “twist‐ring”‐concept as seen on salt shakers, Figure 2d). This specific bypass configuration was chosen such that the resistance of the XM foam, when the bypass is opened, drops to the breathing resistance similar to the breathing resistance of an XF foam in the closed cassette. The dimensions of the cassettes were chosen such that the cassettes closely fitted the HME foams.

### 2.2 Equipment

The pressure drop (a measure of the HME's breathing resistance) of the HME devices was assessed with a digital pressure indicator (DPI 705, BHGE Druck, Houston, Texas) at different airflow rates of 30, 60, and 90 L/min in correspondence with the ISO standards (see Table 1), representing approximately breathing at rest and during light and strenuous exercise.^9^

Performance measurements, measuring the HME's Water Exchange, were executed as validated by van den Boer et al. (2013 and 2014).^14^, ^22^ The measurement protocol was slightly adapted to fit the objectives of this study (see *Study design*). Summarizing, a healthy volunteer breathes through a spirometer set‐up with a standardized breathing pattern, with the HME device connected to a coupler on the other side of the spirometer (Flowhead MLT300 AD Instruments GmbH, Oxfordshire, United Kingdom). First, the HME is conditioned toward its equilibrium water saturation (duration of conditioning is determined separately for each HME). After this initial conditioning, a sequence of weight measurements is conducted, alternating at the end of an inhalation and the end of an exhalation, to determine the HME's Water Exchange. The weight changes of the HME device are measured using a microbalance (Sartorius MC210p, Göttingen, Germany). The HME foam is reconditioned for at least five breathing cycles between each weight measurement. During the measurement sequence, the ambient humidity and temperature of the room are recorded by a commercial humidity sensor (Testo BV, Almere, The Netherlands) to perform data normalization. At the start and end of each measurement sequence, the ambient humidity and temperature of the room is additionally monitored with a hygrometer (Philips Thermo + Hygro, Eindhoven, The Netherlands) and digital thermometer (ThermaLite Digital, E.T.I. Ltd., Worthings, UK) and the temperature of the volunteer is measured with an electronic ear thermometer (Braun WelchAllyn, Kaz Inc., Marlborough, Massachusetts). In this set‐up the volunteer functions as an “artificial lung”. The temperature of the volunteer is used for normalization (see *Analysis*). The volunteer was asked to breath in a fixed rectangular breathing pattern, which is guarded by the spirometer.

### 2.3 Study design

For this study, resistance (Pressure Drop) and humidification performance (Water Exchange) measurements were conducted for 10 XM foams (one batch, batch year: 2019) and 15 XF foams (three batches, batch years: 2017, 2018, and 2019) inside the two different cassette types: both the XF and XM foams in the closed cassette and the XM foams in the cassette with the bypass (Figure 2). All performance measurements were performed by one healthy volunteer (female, 27 years old, ML) for one breathing pattern under room climate conditions. A tidal volume (*V*_*T*_) of 1 L and target flow of 0.33 L/s was chosen, which was a comfortable breathing pattern for the volunteer and corresponds to the ISO standards (see Table 1). After initial conditioning of the HME foam, a sequence of 15 weight measurements was conducted (starting and ending with an exhalation). This resulted in 13 weight changes per HME since the first measurement was disregarded to account for differences in conditioning periods between the HME devices.

### 2.4 Analysis

All performance measurements were normalized to the reference ambient humidity of 5 mg/L and a reference humidity at the tracheal side of 32 mg/L (see Appendix B).^22^ An independent sample *t* test was conducted using IBM SPSS Statistics 25 (SPSS, Chicago, IL) to compare the average performances of the different HME devices.

## 3 RESULTS

**FIGURE 3:**
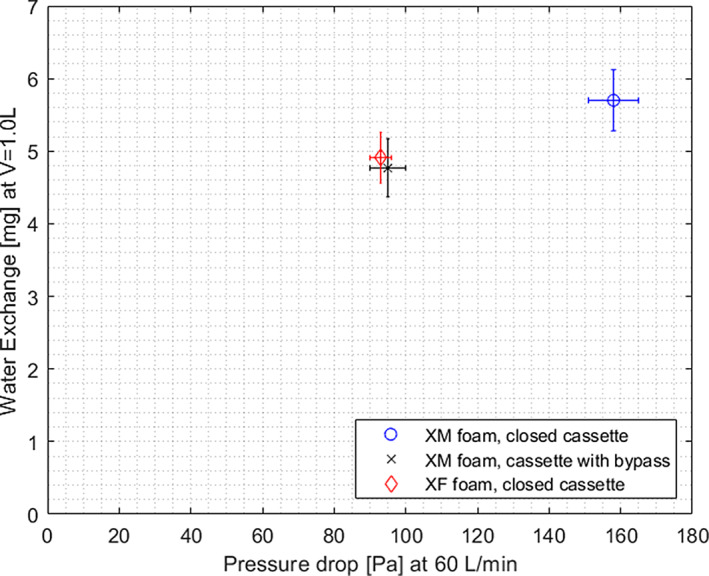
Resistance (Pressure Drop at 60 L/min) against normalized humidificationperformance (Water Exchange at *V*_*T*_ = 1 L) of the different HME devices. The horizontal and vertical error bars indicate the standard deviations from the average Resistance and Water Exchange, respectively. Abbreviations: HME, heat and moisture exchanger; XF, lower resistance/lesser humidification HME; XM, high resistance/high humidification HME; *V_T_*, tidal volume [Color figure can be viewed at wileyonlinelibrary.com]

An overview of the average resistance (Pressure Drop) and the humidification performance (Water Exchange) of all XF and XM foams in the two different HME cassette types are shown in Table 2 and Figure 3.

**TABLE 2:** Overview of the average resistance (pressure drop) and normalized humidification performance (water exchange) of the XM and XF foams in the two different cassette types.

HME device		Pressure Drop in Pa (SD)			Water Exchange in mg (SD)
HME foam type	HME cassette type	At 30 L/min	At 60 L/min	At 90 L/min	At *V*_*T*_ = 1 L, *F* = 0.33 L/s, *AH_amb‐ref_* = 5 mg/L, and *AH_*ts*_* = 32 mg/L
XM foam	Closed cassette	50 (2)	158 (7)	325 (13)	5.70 (0.42)
	Cassette with bypass	29 (1)	95 (5)	201 (11)	4.77 (0.40)
XF foam	Closed cassette	26 (1)	93 (3)	196 (4)	4.91 (0.35)

*Note*: The tidal volume (*V*_*T*_) and airflow rates of the pressure drop measurements correspond to the ISO standards (see Table 1). The different airflow rates of 30, 60, and 90 L/min represent approximately breathing at rest and during light and strenuous exercise.^9^ The SDs of the Water Exchange measurements of the HME devices are comparable to those previously reported by van den Boer et al. (2013).^22^ For the XF foam, a weighted mean and SD were calculated to represent the three different batches in equal proportion.

Abbreviations: *AH_amb‐ref_*, reference ambient humidity; *AH_ts_*, reference humidity at the tracheal side of the HME; *F*, flow; HME, heat and moisture exchanger; *V_T_*, tidal volume; XF, lower resistance/lesser humidification HMESD, standard deviation; XM, high resistance/high humidification HME.

In the closed cassette, the average pressure drops and Water Exchange values of the XM foam are higher than that of the XF foam. When the bypass was introduced in the XM foam's cassette, the pressure drop of the XM foam decreased to a pressure drop similar to the XF foam in the closed cassette. The average Water Exchange of the XM foam in the cassette with bypass was slightly lower than the average Water Exchange of the XF foam in the closed cassette (not significant, *P* > .05). Compared to the XM foam in the closed cassette, the bypass resulted in pressure drop of approximately 60% the original pressure drop value, thus a 40% decrease in resistance, whereas the humidification performance was maintained at approximately 80% of the original Water Exchange value of the XM foam.

## 4 DISCUSSION

This proof of principle study shows that introducing a bypass in the base of an HME cassette can substantially decrease the resistance of a high resistance/high humidification HME (XM) foam to the lower breathing resistance of a lower resistance/lesser humidification HME (XF) foam in the closed cassette, while humidification performance stays at an acceptable level.

Intuitively, one would expect that creating holes in an HME cassette (which lets the air bypass the HME's foam) will decrease the HME's resistance and consequently its humidification performance to a level where the HME will become “useless” for the pulmonary rehabilitation of laryngectomized patients. However, both the theory stating the (almost) quadratic relationship between pressure and flow (Appendix A), as the results of this study indicate that a bypass will decrease the resistance much more than the humidification performance. Additionally, careful examination of existing HMEs shows that the cassettes already (coincidentally) have “bypasses” in their designs and still these HMEs have good Water Exchange values.^14^ For example, the Provox^®^ Luna^®^ HME (Atos Medical AB, Malmö, Sweden) clearly has two side openings acting as “bypasses.”

In this proof of principle study, we used a cassette without speaking valve. However, cassettes without a speaking valve are nowadays often not acceptable to patients with a voice prothesis.^15^ In Appendix B.4, Table B2, a comparison is made between the performance measurements found in this study (Table 2), with the humidification performance values of with the HMEs with speaking valve found by van den Boer et al. (2014a, 2014b) and the manufacturer's specifications (Table 1).^14^, ^23^ Additionally, unpublished experiments' results were included in Table B2, performed in the Netherlands Cancer Institute – Antoni van Leeuwenhoek during the past 3 years. The humidification performance results with and without speaking valve are very similar. Therefore, we predict that a final prototype with speaking valve will have a similar clinically acceptable humidification performance. The assessment of the user functionality and compliance, important device considerations for a final prototype with speaking valve, requires the support of a manufacturer and was outside the scope of this study. Such a study with laryngectomized patients, in which the effectiveness of the final prototype is evaluated, is recommended as the next step.

This proof of principle shows that an adjustable HME is feasible. Such an HME would have several important advantages. In the first place, it can be used by the laryngectomized patients to modify the breathing resistance, which eliminates the need to remove or switch HME types based on activity level. Even if the novel HME cassette is used solely on the lowest resistance setting, it still has a clinically acceptable humidification performance similar to an XF. If laryngectomized patients are not able or willing to switch HMEs, an adjustable HME enables a lower breathing resistance during physical activity and an optimal HME with a higher breathing resistance during nonstrenuous activities. Furthermore, since clinically acceptable breathing resistance does not only vary between physical activity levels but also between laryngectomized patients^1^, ^10^, this novel HME cassette concept could also be employed to gradually train laryngectomized patient to a (higher) HME resistance over time (eg, by using the “twist‐ring” in an intermediate setting). Altogether, this might increase overall HME compliance and pulmonary rehabilitation in laryngectomized patients.

## 5 CONCLUSION

By introducing a bypass, this novel HME cassette prototype allows adjustment between substantially different HME resistances while maintaining appropriate humidification performances. The advantage of the specific bypass in the prototype is that it can easily be opened, closed or adjusted by the laryngectomized patient. This potentially facilitates physical exercise without changing or removing the HME and might therefore increase overall patient compliance.

Currently, this adjustable “bypass”‐principle is not yet available in any commercial HME cassette. We hope that this prototype will be developed further into an effective medical device.

## CONFLICT OF INTEREST

Atos Medical AB had no role in the concept, study design, and drafting of this manuscript. The authors have no other funding, financial relationships, or conflicts of interest to disclose.

## Figures and Tables

**FIGURE A1:**
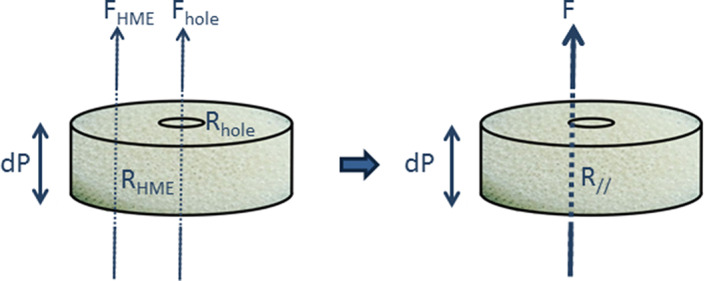
Combining two resistances into one parallel resistance for an HME foam with a hole, a “bypass,” through the center. *dP* is the pressure difference over the HME, *F* the total flow (volume/time), *F*_HME_ the flow through the foam, *F*_Hole_ the flow through the hole [Color figure can be viewed at wileyonlinelibrary.com]

**FIGURE B1:**
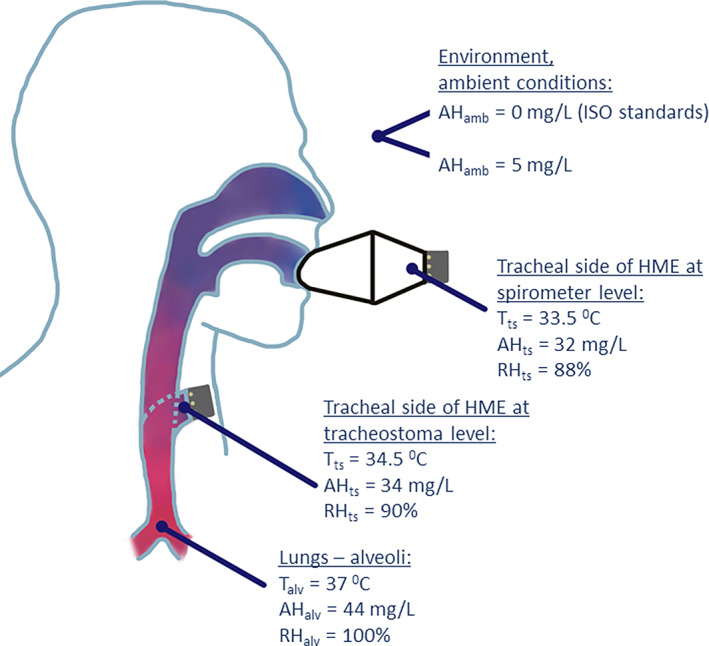
Humidity and temperature values of the upper and lower respiratory tract. For abbreviations, see nomenclature in Appendix B [Color figure can be viewed at wileyonlinelibrary.com]

**FIGURE B2:**
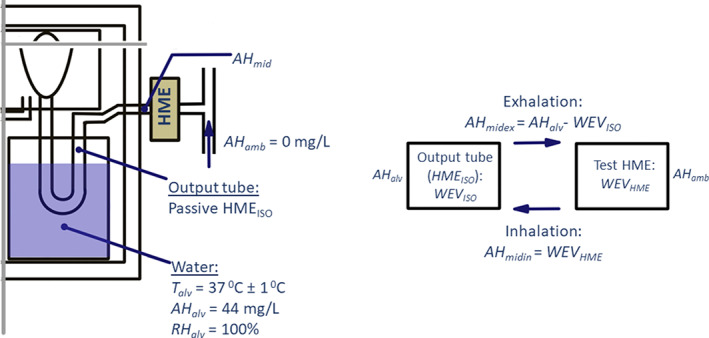
Left: Schematic of the ISO rig (“artificial lung”), in accordance with ISO 9360‐2:2001,^6^ with the test HME placed on the right hand side. The output tube is considered as a (passive) *HME*_*ISO*_. Right: Simplified model of the ISO rig and relations between the equilibrium *WEV*_*ISO*_ and *WEV*_*HME*_ values during inhalation and exhalation of the artificial lung. For abbreviations, see nomenclature in Appendix B [Color figure can be viewed at wileyonlinelibrary.com]

## Data Availability

The data that support the findings of this study are available from the corresponding author upon reasonable request.

## Introduction

In this appendix, we derive the theoretical impact of a bypass (eg, through the center of an HME, Figure A1) on resistance and humidification performance (Water Exchange) of the HME.

For the calculation of resistance, we consider the HME as the combination of the remaining foam and the hole. For the calculation of humidification performance (Water Exchange), we use the remaining foam only.

## Derivation of parallel resistance for a power law relationship between air flow and pressure difference

Analogous to the derivation of parallel electrical resistance using Ohm's law, we can derive the “parallel” resistance (*R*_*//*_) of the resistance remaining foam of the HME (*R*_HME_) and the resistance of the hole (*R*_Hole_) (Figure A1).^1^

Using a power law relationship with an exponent *a*, the following equations apply:

(1) $$\mathrm{dP}=R_{\mathrm{HME}}*{F_{\mathrm{HME}}}^{a}$$

(2) $$\mathrm{dP}=R_{\text{Hole}}*{F_{\text{Hole}}}^{a}$$

(3) $$F=F_{\mathrm{HME}}+F_{\text{Hole}}$$

(4) $$\mathrm{dP}=R_{//}*F^{a}.$$

### Nomenclatures

*dP*: pressure difference over the HME (Pa) *F*: combined flow (L/s)*R*: resistance (Pa/[m^3^/s]^a^)^1^

*R*_*//*_: combined resistance of the parallel resistances *R*_HME_ and *R*_Hole_ (Pa/[m^3^/s]^a^)

Verkerke et al. (2001) found a mixture of a linear and a quadratic relationship and discussed the theoretical background of the linear and quadratic terms.^2^ For the HMEs used in this study, the linear term is small so that the pressure data can be fitted with a power law with an exponent *a* of about 1.8.^1^ From Equation (1) it follows that for a flow (*F*) of 1 L/s (60 L/min), the *dP* (in Pa) is numerically equal to the resistance.

Combining Equations (1) and (2) yields:

(5) $${F_{\mathrm{HME}}}^{a}=\frac{R_{\text{Hole}}}{R_{\mathrm{HME}}}\;{F_{\text{Hole}}}^{a}\;\text{or}\;F_{\mathrm{HME}}={\left(\frac{R_{\text{Hole}}}{R_{\mathrm{HME}}}\right)}^{1/a}*F_{\text{Hole}}$$

Combining Equations (3) and (4) yields: *R*_*//*_ = *dP/F* ^*a*^ = *dP/*(*F*_HME_ + *F*_Hole_)^a^. Using Equation (2) followed by Equation (5) yields the resistance of the remaining foam and hole together:

(6) $$R_{//}=\frac{R_{\text{Hole}}*F_{\text{Hole}}^{a}}{{\left(F_{\mathrm{HME}}+F_{\text{Hole}}\right)}^{a}}=\frac{R_{\text{Hole}}*F_{\text{Hole}}^{a}}{{\left(F_{\text{Hole}}*{\left(\frac{R_{\text{Hole}}}{R_{\mathrm{HME}}}\right)}^{1/a}+F_{\text{Hole}}\right)}^{a}}=\frac{R_{\text{Hole}}}{{\left({\left(\frac{R_{\text{Hole}}}{R_{\mathrm{HME}}}\right)}^{1/a}+1\right)}^{a}}=\frac{R_{\text{Hole}}*R_{\mathrm{HME}}}{{\left({\left(R_{\text{Hole}}\right)}^{1/a}+{\left(R_{\mathrm{HME}}\right)}^{1/a}\right)}^{a}}$$

For *a* = 2, this simplifies to:

$$R_{//}=\frac{R_{\mathrm{HME}}*R_{\text{Hole}}}{{\left(\sqrt{R_{\mathrm{HME}}}+\sqrt{R_{\text{Hole}}}\right)}^{2}}$$

## Relation between resistance and cross sectional area

For a homogeneous air stream (assuming a homogeneous flow profile) inside a homogeneous HME, the flow (*F*) is proportional to the cross sectional area (*A*). Combining this with Equations (1) and (2) gives:

(7) $$\frac{R\left(A_{1}\right)}{{A_{1}}^{a}}=\frac{R\left(A_{2}\right)}{{A_{2}}^{a}}$$

For *a* = 2, this means that resistance is proportional to the inverse square of the area, so with the inverse of the fourth power of the radius of a cylindrical HME. Consequently, resistances decrease very quickly if the HME's radius is enlarged.

## Performance

Performance, defined as the Water Exchange, is in first order proportional to the volume of air passing through the HME (*F*_HME_) and to the mass of remaining foam.^3,4^

Combining Equations (1) and (4), we can calculate the flow through the remaining foam *F*_HME_ (and similarly from Equations (1) and (3) the flow through the hole, *F*_Hole_):

(8) $$F_{\mathrm{HME}}=F*{\left(\frac{R_{//}}{R_{\mathrm{HME}}}\right)}^{1/a}$$

## Example: Calculated impact of a bypass on the resistance and performance of an HME

For the example we use the results from Table 2. The high resistance—high performance HME (the XM foam in the closed cassette, Figure 2) has a Water Exchange of 5.70 mg and a resistance of 157. The resistance was determined by fitting the three pressure drop measurements (Table 2) to Equation (1) using *a* = 1.8, which is approximately numerically equal to the pressure drop at 60 L/min. The measured resistances (pressure drops) of our HME devices (Table 2) are substantially lower than those of the clinical XM and XF HME device (Table 1) due to the absence of a speaking valve in our HME cassette designs. Nonetheless, the relative pressure drop between the XM and XF foam is similar (XF/XM = 0.54 with speaking valve and 0.59 without speaking valve).

The XM foam is cylindrical and has a diameter of 21 mm and a height of 11 mm. In this HME foam, we introduce a bypass, for instance a simple hole with a diameter of about 4 mm in the middle of the HME foam (which has approximately the same effect on the HME's resistance as the type of bypass used in the Main paper). The area of this hole is 3% of the area of the XM foam. The remaining 97% of the foam will thus have a resistance of 166 (Equation 7). If we assume a resistance of about 1000 for this hole (based on pressure drop values measured over small pipes), we can calculate with Equation (6) that the HME with bypass will have the intended resistance of 94 (similar to that of the “low resistance” reference HME, the XF foam in the closed cassette). The resistance has thus decrease to 60% of the original resistance value. Using Equation (8) and the knowledge of the first order proportionality between the HME's performance and the volume of air passing through the HME and the mass of the remaining foam, we find that the performance is reduced to approximately 73% of the original performance value, corresponding to Water Exchange of 4.2 mg Water Exchange.^*^

^*^We expect the performance of this HME with bypass (hole through its foam) to be slightly higher than calculated in this example; during experiments we have made the observation that Water Exchange increases when local air speed (not to be confused with total air flow) decreases, but the order of this secondary effect is to be further investigated. This flow dependence was not taken into account in previous studies (van den Boer et al. [2014a, 2014b]).^3,4^

1.Walker IS, Wilson DJ, Sherman MH. A comparison of the power law to quadratic formulations for air infiltration calculations. *Energy and Buildings*. 1997;27(3):293‐299.

2.Verkerke G, Geertsema A, Schutte H. Airflow Resistance of Airflow‐Regulating Devices Described by Independent Coefficients. *The Annals of otology*, *rhinology*, *and laryngology*. 2001;110:639‐645.

3.van den Boer C, Muller SH, Vincent AD, van den Brekel MW, Hilgers FJ. Ex vivo assessment and validation of water exchange performance of 23 heat and moisture exchangers for laryngectomized patients. *Respiratory Care*. 2014; 59(8):1161–1171.

4.van den Boer C, Muller SH, Vincent AD, Züchner K, van den Brekel MW, Hilgers FJ. A novel, simplified ex vivo method for measuring water exchange performance of heat and moisture exchangers for tracheostomy application. *Respiratory care*. 2013;58(9):1449‐1458.

## Nomenclatures

*AH*: Absolute Humidity (mg/L)

*HME*: Heat and Moisture Exchanger

*ML*: Moisture Loss (mg/L)

*RH*: Relative Humidity (%)

*V*_*T*_: Tidal volume (L)

*WE*: Water Exchange (mg)

*WEV*: Water Exchange per *V*_*T*_ (mg/L)

Subscripts:

_*1*_: standardized conditions for normalization

_*2*_: conditions during the performance measurement

_*alv*_: “alveolar”

_*amb*_: ambient conditions

_*HME*_: test HME, measured

_*HMEcalc*_: test HME, calculated

_*ISO*_: output tube of the ISO rig

_*midex*_: the position between the ISO rig and test HME during exhalation

_*midin*_: the position between the ISO rig and test HME during inhalation

_*ts*_: on the tracheal side of the HME

Even though Water Exchange (*WE*) and Moisture Loss (*ML*) are both a measure of the HME's humidification performance (the amount of water recovered by an HME), comparison between the two performance measures is complicated. Water Exchange is measured *in vivo*, whereas Moisture Loss is measured *ex vivo*. Moreover, the performance of an HME depends on the *AH*_*amb*_ and *AH*_*ts*_ during the measurement, and on *V*_*T*_. This appendix described the steps required for a reliable comparison between *WE* and *ML*:

- Normalization of *WE* to standardized *AH*s: *AH*_*amb*_ and *AH*_*ts*_ (Appendix B.1).
- Conversion between different values of *V*_*T*_ (Appendix B.2)
- Theory: conversion between *WE* and *ML* (Appendix B.3)
- Comparison of *WE* and *ML* values for this study (Appendix B.4)

## B.1. Normalization of Water Exchange to standardized AHs (*AH_amb_* and *AH_ts_*)

The performance (defined as Moisture Loss *ML* or Water Exchange *WE*) of an HME depends on the absolute humidities (*AH*s) on both sides of the HME; on the *AH* at the tracheal side of the HME (*AH*_*ts*_) and on the ambient *AH* (*AH*_*amb*_). Therefore, the measured *WE* must be normalized and converted to standardized conditions to be able to compare measurements under different conditions.

Normalization of the *WE* data between different values of *AH*_*ts*_ and *AH*_*amb*_ is done using a generalized equation of van den Boer et al. (2013):

(9) $$\text{WE}\;\left(@{\mathrm{AH}}_{\text{ts1}}\;\text{and}\;{\mathrm{AH}}_{\text{amb1}}\right)=\text{WE}\;\left(@{\mathrm{AH}}_{\text{ts2}}\;\text{and}\;{\mathrm{AH}}_{\text{amb2}}\right)*\frac{\left({\mathrm{AH}}_{\mathrm{ts}1}-{\mathrm{AH}}_{\mathrm{amb}1}\right)}{\left({\mathrm{AH}}_{\mathrm{ts}2}-{\mathrm{AH}}_{\mathrm{amb}2}\right)}$$

### B.1.1. Standardized ambient Absolute Humidity (*AH_amb_*)

Water Exchange is specified by van den Boer et al. (2013, 2014a, and 2014b, Table 1) at a low but realistic *AH*_*amb*_ of 5 mg/L, which we also used in this study as the standardized *AH*_*amb*_ to normalized the HMEs' performance to (except in Appendix B.3, Table B1).

**TABLE B1:** Normalized input and verification data of different HMEs for the determination of the conversion from the normalized Water Exchange (*WE*) to Moisture Loss (*ML*).

All values in mg/L		${\mathrm{WEV}}_{\mathrm{HME}}^{32;0}$	*ML_HME_*	*ML_HMEcalc_*	Ref. for *WE_HME_*
Year	HME type	@32; 0 mg/L (*V*_*T*_ = 1 L)	@44; 0 mg/L (*V*_*T*_ = 1 L)	@44; 0 mg/L (*V*_*T*_ = 1 L)	
2014	Hiflow	4.49	24.4	23.9	Van den Boer et al. (2014b)^2^
2014	Normal	4.39	23.7	23.9	Van den Boer et al. (2014b)^2^
2014	XtraFlow	4.49	24	23.9	Van den Boer et al. (2014b)^2^
2014	XtraMoist	7.58	21.5	22.1	Van den Boer et al. (2014b)^2^
2014	Hiflow	4.44	24.4	23.9	Van den Boer et al. (2014a)^1^
2014	Normal	5.37	23.7	23.4	Van den Boer et al. (2014a)^1^
2014	XtraFlow	5.92	24	23.1	Van den Boer et al. (2014a)^1^
2014	XtraMoist	7.09	21.5	22.4	Van den Boer et al. (2014a)^1^
2016	XtraFlow	5.01	24	23.6	^a^
2016	XtraMoist	6.79	21.5	22.6	^a^
2017	XtraFlow	4.41	24	23.9	^a^
2017	XtraMoist	5.58	21.5	23.3	^a^

*Note*: Normalized *WEV_HME_* (${\mathrm{WEV}}_{\mathrm{HME}}^{32;0}$) values at *V*_*T*_ = 1 L were calculated (see Appendix B.1 and B.2) from the values as measured by van den Boer et al. (2014a, 2014b).^1,2^ *ML_HME_* values were provided by the manufacturer (Atos Medical, Malmö, Sweden) in accordance with ISO 9360‐2:2001.^3^ *ML_HMEcalc_* was calculated from *WE_HME_* using Equation (21) and ${\mathrm{WEV}}_{\mathrm{ISO}}^{44;0}$= 17.8 mg. For, abbreviations, see nomenclature in Appendix B.3.

*Note*: 1. van den Boer C, Muller SH, Vincent AD, van den Brekel MW, Hilgers FJ. Ex vivo assessment and validation of water exchange performance of 23 heat and moisture exchangers for laryngectomized patients. *Respiratory Care*. 2014; 59(8): 1161‐1171.

*Note*: 2. van den Boer C, Muller SH, Vincent AD, Züchner K, van den Brekel MWM, Hilgers FJM. Ex vivo water exchange performance and short‐term clinical feasibility assessment of newly developed heat and moisture exchangers for pulmonary rehabilitation after total laryngectomy. *European Archives of Oto‐Rhino‐Laryngology*. 2014;271(2):359‐366.

*Note*: 3. International Standards Organization. Anesthetic and respiratory equipment—heat and moisture exchangers (HMEs) for humidifying respired gases in humans. HMEs for use with tracheostomized patients having minimal tidal volume of 250 mL. Geneva: ISO; 9360‐2:2001.

a The observations in 2016 and 2017 were made following the protocol of van den Boer (internal communication).

### B.1.2. Standardized Absolute Humidity at the tracheal side of the HME (*AH_ts_*)

Using the data from Scheenstra et al. (2010 and 2011)^4,5^ measured in laryngectomized patients, we estimate that for laryngectomized patients the humidity on the tracheal side of the HME at tracheostoma level (1 cm behind the HME) is 34 mg/L at 34.5°C (*RH* = 90%, see Figure B1).^†^ The HMEs' Water Exchange data in our study and that of van den Boer et al. (2014a and 2014b) were measured with volunteers, with the HME connected to the volunteer's mouth using a spirometer.^2,3^ This means that the temperature and humidity at the tracheal side of HME at spirometer level are slightly lower than at tracheostoma level. Comparing temperature observations made during volunteer experiments with those made in laryngectomized patients, we estimate that the humidity at the tracheal side of the HME at spirometer level is 32 mg/L at 33.4°C (*RH* = 88%, see Figure B2).^‡^ In this volunteer study, we therefore normalized the HMEs' performance data to a humidity of 32 mg/L at the tracheal side of the HME at spirometer level (*AH*_*ts*_).

### B.1.3. Converting Water Exchange values between volunteers and patients

Water Exchange values measured in volunteers (specified at *AH*_*ts*_ = 32 mg/L and *AH*_*amb*_ = 5 mg/L) can be converted to the *WE* values which would be found in laryngectomized patients (specified at *AH*_*ts*_ = 34 mg/L and *AH*_*amb*_ = 5 mg/L) using Equation (9). These values will be (34‐5)/(32‐5) = 7% higher.

## B.2. Conversion for tidal volume (*V*_*T*_)

The HME's Water Exchange *WE* strongly depends on the *V*_*T*_, because in the first order the amount of water vapor that can be condensed or evaporated will be proportional to the volume of air that goes through the HME. Usually, the HME's *WE* is reported at *V*_*T*_ = 0.5 L, which is comparable to the *V*_*T*_ of a laryngectomized patient at rest (Table 1, Main Paper). Manufacturers often specify the HME's *ML* only at a *V*_*T*_ of 1.0 L.^§^ When comparing the HME's *WE* (mg) and *ML* (mg/L), we first have to convert *WE* to the *WE* per Tidal Volume:

(10) $$\mathrm{WEV}=\frac{\text{WE}}{V_{T}}$$

However, neither *WEV* nor *ML* is independent of *V*_*T*._ When *V*_*T*_ increases, *WE* increases less than linear with *V*_*T*_ (see, for example, fig. 2 in Van den Boer et al. 2014a)^3^, thus *WEV* decreases with increasing *V*_*T*_. The ISO norm^**^ also assumes that *ML* may depend on *V*_*T*_. Therefore, *WEV* and *ML* must be compared at the same *V*_*T*._ *WEV* at *V*_*T*_ = 0.5 L has been converted to *WEV* at *V*_*T*_ = 1.0 L using the *WE* data fits as a function of volume (see Van den Boer et al. [2014a], fig. 2 and Appendix 2). To avoid additional conversions, we have chosen to perform our Water Exchange measurements in this study directly at *V*_*T*_ = 1.0 L.

## B.3. Comparison of *in vivo* Water Exchange and *ex vivo* Moisture Loss

*WEV* and *ML* have the same unit (mg/L), but the comparison of the HMEs' *WEV* values and *ML* values as specified by the manufacturer (in accordance with ISO 9360‐2:2001)^6^ is still complicated. *WE* and *WEV* values are measured *in vivo* in volunteers and laryngectomized patients. In a human, the trachea is an active (heated and moisture providing) HME, and thus provides a constant humidity on the tracheal side of the HME. In contrast, *ML* values are measured *ex vivo* with an artificial lung, the ISO rig. The “trachea” output tubing in the artificial lung is a less efficient passive HME. Therefore, the Absolute Humidity (*AH*) at the tracheal side of the HME is not constant; it will increase when a higher performance test‐HME is placed in this ISO rig, and vice versa, and will thus influence the tested HME's performance results.

Using a compartment model of the ISO rig (see Figure B2, B.3.1 and B.3.2) we can derive the relationship between *ML* and *WEV* (see B.3.4).

Unfortunately, the *WEV* value of the output tube of the ISO rig is not specified, so we need an additional step to determine this value. Using the *WE* values of different HMEs for which also the *ML* values are known, the *WEV* of the output tube can be determined (see B.3.3).

### B.3.1. Properties of the ISO rig

The ISO rig maintains water at 37°C, so the “alveolar” absolute humidity (*AH*_*alv*_) is 44 mg/L. The ambient absolute humidity (*AH*_*amb*_) is 0 mg/L. We consider the output tube of the ISO rig as a (passive) *HME*_*ISO*_ with Water Exchange ${\mathrm{WEV}}_{\mathrm{ISO}}^{44;0}$ if no test HME is present on the ISO rig (the superscripts denote the humidities on either side of the output tube). The test HME has a measured *WEV*_*HME*_ and a normalized Water Exchange ${\mathrm{WEV}}_{\mathrm{HME}}^{32;0}$ (see also Appendix B.1). We normalize the *WEV*_*HME*_ values to an *AH*_*amb*_ of 0 mg/L in accordance with the ISO standards (instead of 5 mg/L as used in the clinical articles^1‐3^), to enable an easy conversion between Water Exchange and Moisture Loss values (see Appendix B.3.4).

When the test HME is mounted on the ISO rig, the *WEV* values of the output tube and test HME adapt and a dynamic equilibrium situation is established. Figure B2, right image, shows a model of the relations between the equilibrium *WEV*_*ISO*_ and *WEV*_*HME*_ values during inhalation and exhalation of the artificial lung.

### B.3.2. Basic equations of the ISO rig model

During inhalation, the test HME increases the Absolute Humidity of the incoming airstream by *WEV*_*HME*_. In ISO conditions, the *AH*_*amb*_ is equal to 0 mg/L (see Figure B2), thus:

(11) $${\mathrm{AH}}_{\text{midin}}={\mathrm{AH}}_{\mathrm{amb}}+{\mathrm{WEV}}_{\mathrm{HME}}={\mathrm{WEV}}_{\mathrm{HME}}$$

The Moisture Loss of the test HME is: *ML_HME_* = *AH_midex_* − *AH_midin_*= *AH_midex_* − *WEV_HME_* (see Figure B2), which can be rewritten to:

(12) $${\mathrm{WEV}}_{\mathrm{HME}}={\mathrm{AH}}_{\text{midex}}–{\mathrm{ML}}_{\mathrm{HME}}$$

In the equilibrium situation during exhalation, the output tube *HME*_*ISO*_ reduces the Absolute Humidity of the outgoing airstream of the ISO rig by *WEV*_*ISO*_ (see Figure B2). Thus *AH*_*midex*_ = *AH*_*alv*_ − *WEV*_*ISO*_, and with *AH*_alv_ = 44 mg/L:

(13) $${\mathrm{WEV}}_{\mathrm{ISO}}=44–{\mathrm{AH}}_{\text{midex}}$$

Using the normalization equation for the Water Exchange from Appendix B.1 (Equation 9), we find:

(14) $${\mathrm{WEV}}_{\mathrm{ISO}}={\mathrm{WEV}}_{\mathrm{ISO}}^{44;0}*\frac{\left(44-{\mathrm{WEV}}_{\mathrm{HME}}\;\right)}{44}$$

(15) $${\mathrm{WEV}}_{\mathrm{HME}}={\mathrm{WEV}}_{\mathrm{HME}}^{32;0}*\frac{{\mathrm{AH}}_{\text{midex}}}{32}$$

### B.3.3. Determination of ${\mathrm{WEV}}_{\mathrm{ISO}}^{44;0}$

Unfortunately, ${\mathrm{WEV}}_{\mathrm{ISO}}^{44;0}$ is not specified for the ISO test rig. However, if both the normalized *ML*_*HME*_ and ${\mathrm{WEV}}_{\mathrm{HME}}^{32;0}$ values of a reference HME are known, we can determine ${\mathrm{WEV}}_{\mathrm{ISO}}^{44;0}$ as a function of *ML*_*HME*_ and ${\mathrm{WEV}}_{\mathrm{HME}}^{32;0}$, by eliminating the unknown variables *WEV*_*ISO*_, *WEV*_*HME*_, and *AH*_*midex*_ from Equations (12) to (15).

Eliminate *WEV*_*ISO*_ by combining (14) and (15):

(16) $$44-{\mathrm{AH}}_{\text{midex}}={\mathrm{WEV}}_{\mathrm{ISO}}^{44;0}*\frac{\left(44-{\mathrm{WEV}}_{\mathrm{HME}}\right)}{44}$$

Eliminate *WEV*_*HME*_ by substituting Equation (15) into Equation (16):

(17) $$44–{\mathrm{AH}}_{\text{midex}}={\mathrm{WEV}}_{\mathrm{ISO}}^{44;0}*\left(44–\left({\mathrm{WEV}}_{\mathrm{HME}}^{32;0}*{\mathrm{AH}}_{\text{midex}}/32\right)\;\right)/44$$

Combining Equations (12) and (15):

$${\mathrm{AH}}_{\text{midex}}–{\mathrm{ML}}_{\mathrm{HME}}={\mathrm{WEV}}_{\mathrm{HME}}^{32;0}*\frac{{\mathrm{AH}}_{\text{midex}}}{32}$$

(18) $$\text{Rewrite}:{\mathrm{AH}}_{\text{midex}}=\frac{ML_{\mathrm{HME}}}{1-\frac{{\mathrm{WEV}}_{\mathrm{HME}}^{32;0}}{32}}$$

Eliminate *AH*_*midex*_ by substituting Equation (18) into Equation (17) and multiplying both sides by (1‐ ${\mathrm{WEV}}_{\mathrm{HME}}^{32;0}$/32)*44*32:

$$44*\left(44*32–44*{\mathrm{WEV}}_{\mathrm{HME}}^{32;0}–32*{\mathrm{ML}}_{\mathrm{HME}}\right)={\mathrm{WEV}}_{\mathrm{ISO}}^{44;0}*(44*32-{\mathrm{WEV}}_{\mathrm{HME}}^{32;0}–\left({\mathrm{WEV}}_{\mathrm{HME}}^{32;0}*{\mathrm{ML}}_{\mathrm{HME}}\;\right)$$

Rewriting yields ${\mathrm{WEV}}_{\mathrm{ISO}}^{44;0}$ as a function of ${\mathrm{WEV}}_{\mathrm{HME}}^{32;0}$ and *ML*_*HME*_:

(19) $${\mathrm{WEV}}_{\mathrm{ISO}}^{44;0}=\frac{44*\left(44*32-44*{\mathrm{WEV}}_{\mathrm{HME}}^{32;0}-32*{\mathrm{ML}}_{\mathrm{HME}}\right)}{\left(44*32-44*{\mathrm{WEV}}_{\mathrm{HME}}^{32;0}-{\mathrm{ML}}_{\mathrm{HME}}*{\mathrm{WEV}}_{\mathrm{HME}}^{32;0}\right)}$$

Using the ${\mathrm{WEV}}_{\mathrm{HME}}^{32;0}$ values of different HMEs for which also the *ML*_*HME*_ values are known, the ${\mathrm{WEV}}_{\mathrm{ISO}}^{44;0}$ of the output tube can be determined.

In order to minimize the effect of measurement errors in the Water Exchange values,^2,3^ we used all available reference HMEs data for which both the ${\text{WE}}_{\mathrm{HME}}^{32;0}$ and *ML_HME_* values are known: the Water Exchange values as measured by van den Boer et al. (2014a, 2014b,^2,3^ normalized to 32 mg/L; 0 mg/L as described in Appendix B.1), and the Moisture Loss values as specified by the manufacturer, respectively. Table B1 (columns $“{\mathrm{WEV}}_{\mathrm{HME}}^{32;0}”$and “*ML*_*HME*_”) gives an overview of the available data we used. *ML* values are only specified for *V*_*T*_ = 1 L so that ${\mathrm{WEV}}_{\mathrm{ISO}}^{44;0}$ will only be determined for *V*_*T*_ = 1 L.

The ${\mathrm{WEV}}_{\mathrm{HME}}^{32;0}$ values were converted into *ML*_*HMEcalc*_ values using Equation (21) (see below). By using a sum of least squares solver over the difference between the calculated *ML*_*HMEcalc*_ values and the *ML*_*HME*_ values as specified by the manufacturer, the optimal ${\mathrm{WEV}}_{\mathrm{ISO}}^{44;0}$ was determined.

We find that ${\mathrm{WEV}}_{\mathrm{ISO}}^{44;0}$= 17.8 mg/L at *V*_*T*_ = 1 L for the ISO rig as used by the manufacturer Atos Medical (Malmö, Sweden).

### B.3.4. Conversion between Water Exchange and Moisture Loss values

Knowing ${\mathrm{WEV}}_{\mathrm{ISO}}^{44;0}$, Equation (19) can be rewritten to calculate the test HME's *ML*_*HMEcalc*_ from ${\mathrm{WEV}}_{\mathrm{HME}}^{32;0}$:

(20) $${\mathrm{ML}}_{\text{HMEcalc}}=\frac{44*\left(32*{\mathrm{WEV}}_{\mathrm{ISO}}^{44;0}-32*44-{\mathrm{WEV}}_{\mathrm{ISO}}^{44;0}*{\mathrm{WEV}}_{\mathrm{HME}}^{32;0}+44*{\mathrm{WEV}}_{\mathrm{HME}}^{32;0}\;\right)}{\left({\mathrm{WEV}}_{\mathrm{ISO}}^{44;0}*{\mathrm{WEV}}_{\mathrm{HME}}^{32;0}-44*32\right)}.$$

Similarly, Equation (19) can also be rewritten to calculate ${\mathrm{WEV}}_{\text{HMEcalc}}^{32;0}$ if *ML*_*HME*_ is known:

(21) $${\mathrm{WEV}}_{\text{HMEcalc}}^{32;0}=\frac{44*32*\left({\mathrm{WEV}}_{\mathrm{ISO}}^{44;0}-44-{\mathrm{ML}}_{\mathrm{HME}}\;\right)}{\left(44*{\mathrm{WEV}}_{\mathrm{ISO}}^{44;0}+{\mathrm{WEV}}_{\mathrm{ISO}}^{44;0}*{\mathrm{ML}}_{\mathrm{HME}}-44*44\right)}.$$

All values in mg/L and at the same *V*_*T*_ (at which ${\mathrm{WEV}}_{\mathrm{ISO}}^{44;0}$ also must be known).

In this article we use *V*_*T*_ = 1 L and ${\mathrm{WEV}}_{\mathrm{ISO}}^{44;0}$= 17.8 mg/L for the ISO rig of Atos Medical (Malmö, Sweden, see Appendix B.3.3).

## B.4. Comparison between Water Exchange and Moisture Loss values for this study

After normalization to standardized *AH*s and conversion to the appropriate *V*_*T*_, the HME's WEV values can be converted into corresponding the *ML* values, and vice versa using Equations (20) and (21).

In Table B2, a comparison is made between the performance measurements found in this study (Main paper, Table 2), with the performance values of the HMEs found by van den Boer et al. (2014a, 2014b) and the manufacturer's specifications (Main paper, Table 1) for a *V*_*T*_ = 1 L.^2,3^ Furthermore, unpublished experiments' results were included in Table B2, performed in the Netherlands Cancer Institute ‐ Antoni van Leeuwenhoek (NKI‐AVL, Amsterdam, the Netherlands) during the past 3 years. Table B2 shows that overall the performance results, even if variable, are very comparable and the difference in performance with and without the speaking valve, if any, is small.

^†^The values at the tracheostoma level are valid for tidal volumes of about 0.5 to 1.0 L and for HMEs with the typical performance of current HMEs. For HMEs with a much better performance, the temperature and the absolute humidity at the tracheostoma level will be higher. The impact of dead space on AH at the end of inspiration has been neglected.

^‡^The actual body temperature of the volunteer (or laryngectomized patient) will also influence the AH_ts_. We used the measured body temperature of the volunteer (which was stable within 1C) to normalize AH_ts_ to the value corresponding with a body temperature of 37°C. The measured body temperature of the volunteer is corrected with a constant (−3.6°C = 37‐33.4) to estimate the temperature T_ts2_ at spirometer level (see Figure 5). Based on this T_ts2_, the AH_ts2_ is calculated (with a reference RH_ts_ of 88%) and used in Equation (9).

^§^Manufactures specifications are performed in accordance with ISO 9360‐2:2001^6^. ISO 9360‐2:2001 offers the choice to perform the measurements at three different tidal volumes (VT = 0.5, 1.0, and 1.5 L).

**TABLE B2:** Comparison of the data measured in this study with the Water Exchange and Moisture Loss values (in accordance with ISO 9360‐2:2001)^6^ of the HMEs.

All values in mg/L, at *V*_*T*_ = 1 L	Water Exchange/*V*_*T*_, normalized to 32/5 mg/L					Moisture Loss, normalized to 44/0 mg/L	
	This study	Van den Boer et al. (2014a)^3^	Van den Boer et al. (2014b)^2^	NKI‐AVL unpublished 2016	NKI‐AVL unpublished (averaged over 2016, 2017 and 2018)	This study, calculated	Atos Medical
Cassette foam	Narrow fitting, no speaking valve	with speaking valve	with speaking valve	with speaking valve	Narrow fitting, no speaking valve	Narrow fitting, no speaking valve	with speaking valve
XtraMoist	5.70	5.98	6.40	5.73	5.47	22.6	21.5
XtraFlow	4.91	4.99	3.79	4.23	n.a.	23.2	24.0

*Note*: All Water Exchange data from Van den Boer et al.^2,3^ were normalized to *AH_amb_* = 5 mg/L and *AH_ts_ = *32 mg/L (Appendix B.1) and converted to *V*_*T*_ = 1 L (Appendix B.2). Van den Boer et al. measured in volunteers, so the actual *AH_ts_* during the measurements was about 32 mg/L. However, they performed their data normalization with an *AH_ts_* of 44 mg/L (*AH* in the alveoli of the lungs). Using the appropriate *AH_ts_* value, only has a minor impact on the results of van den Boer et al. (2014a and 2014b); the *WE* increases with approximately 4% and the rating of HMEs stays the same.]) Moisture Loss data for our HME devices were determined using Equation (21) (Appendix B.3.4). For comparison with the *ML* values, the table shows *WE/V*_*T*_ values (at *V*_*T*_ = 1 L, numerically equal to *WE*).

Abbreviations: NKI‐AVL, Netherlands Cancer Institute – Antoni van Leeuwenhoek (Amsterdam, The Netherlands); also see nomenclature in Appendix B.

*Note*: 1. International Standards Organization. Anesthetic and respiratory equipment—heat and moisture exchangers (HMEs) for humidifying respired gases in humans. HMEs for use with tracheostomized patients having minimal tidal volume of 250 mL. Geneva: ISO; 9360‐2:2001.

*Note*: 2. van den Boer C, Muller SH, Vincent AD, Züchner K, van den Brekel MWM, Hilgers FJM. Ex vivo water exchange performance and short‐term clinical feasibility assessment of newly developed heat and moisture exchangers for pulmonary rehabilitation after total laryngectomy. *European Archives of Oto‐Rhino‐Laryngology*. 2014;271(2):359‐366.

*Note*: 3. van den Boer C, Muller SH, Vincent AD, van den Brekel MW, Hilgers FJ. Ex vivo assessment and validation of water exchange performance of 23 heat and moisture exchangers for laryngectomized patients. *Respiratory Care*. 2014; 59(8): 1161‐1171.

1. van den Boer C, Muller SH, Vincent AD, Züchner K, van den Brekel MW, Hilgers FJ. A novel, simplified ex vivo method for measuring water exchange performance of heat and moisture exchangers for tracheostomy application. *Respiratory care*. 2013;58(9):1449‐1458.

2. van den Boer C, Muller SH, Vincent AD, Züchner K, van den Brekel MWM, Hilgers FJM. ex vivo water exchange performance and short‐term clinical feasibility assessment of newly developed heat and moisture exchangers for pulmonary rehabilitation after total laryngectomy. *European Archives of Oto‐Rhino‐Laryngology*. 2014;271(2):359‐366.

3. van den Boer C, Muller SH, Vincent AD, van den Brekel MW, Hilgers FJ. ex vivo assessment and validation of water exchange performance of 23 heat and moisture exchangers for laryngectomized patients. *Respiratory Care*. 2014;59(8):1161‐1171.

4. Scheenstra RJ, Muller SH, Vincent A, Sinaasappel M, Hilgers FJ. Influence of breathing resistance of heat and moisture exchangers on tracheal climate and breathing pattern in laryngectomized individuals. *Head & Neck*. 2010;32(8):1069‐1078.

5. Scheenstra RJ, Muller SH, Vincent A, Ackerstaff AH, Jacobi I, Hilgers FJ. A new heat and moisture exchanger for laryngectomized patients: endotracheal temperature and humidity. *Respiratory care*. 2011;56(5):604‐611.

6. International Standards Organization. Anesthetic and respiratory equipment—heat and moisture exchangers (HMEs) for humidifying respired gases in humans. HMEs for use with tracheostomized patients having minimal tidal volume of 250 mL. Geneva: ISO; 9360‐2:2001.
